# The efficacy of stereotactic minimally invasive thrombolysis at different catheter positions in the treatment of small- and medium-volume basal ganglia hemorrhage (SMITDCP I): a randomized, controlled, and blinded endpoint phase 1 trial

**DOI:** 10.3389/fneur.2023.1131283

**Published:** 2023-05-12

**Authors:** Xin Huang, Ziwei Yan, Lai Jiang, Shaojun Chen, Yifei Liu

**Affiliations:** ^1^Department of Neurosurgery, The People's Hospital of China Three Gorges University, The First People's Hospital of Yichang, Yichang, Hubei, China; ^2^Department of Ultrasound Diagnostics, The People's Hospital of China Three Gorges University, The First People's Hospital of Yichang, Yichang, Hubei, China; ^3^Department of Anesthesiology, The People's Hospital of China Three Gorges University, The First People's Hospital of Yichang, Yichang, Hubei, China

**Keywords:** stereotactic, minimally invasive, catheter location, hypertensive cerebral hemorrhage, small to medium volume basal ganglia hemorrhage

## Abstract

**Objective:**

The aim of this study was to evaluate the effects of stereotactic minimally invasive puncture with different catheter placement positions when combined with urokinase thrombolysis for the treatment of small- and medium-volume basal ganglia hemorrhage. Our goal was to identify the best minimally invasive catheter placement position to enhance therapeutic efficacy for patients with cerebral hemorrhage.

**Methods:**

The stereotactic minimally invasive thrombolysis at different catheter positions in the treatment of small- and medium-volume basal ganglia hemorrhage (SMITDCPI) was a randomized, controlled, and endpoint phase 1 trial. We recruited patients with spontaneous ganglia hemorrhage (medium-to-small and medium volume) who were treated in our hospital. All patients received stereotactic, minimally invasive punctures combined with an intracavitary thrombolytic injection of urokinase hematoma. A randomized number table method was used to divide the patients into two groups concerning the location of catheterization: a penetrating hematoma long-axis group and a hematoma center group. The general conditions of the two groups of patients were compared, and the data were analyzed, including the time of catheterization, the dosage of urokinase, the amount of residual hematoma, the hematoma clearance rate, complications, and the National Institute of Health stroke scale (NIHSS) score data at 1 month after surgery.

**Results:**

Between June 2019 and March 2022, 83 patients were randomly recruited and assigned to the two groups as follows: 42 cases (50.60%) to the penetrating hematoma long-axis group and 41 cases (49.40%) to the hematoma center group. Compared with the hematoma center group, the long-axis group was associated with a significantly shorter catheterization time, a lower urokinase dose, a lower residual hematoma volume, a higher hematoma clearance rate, and fewer complications (*P* < 0.05). However, there were no significant differences between the two groups in terms of the NIHSS scores when tested 1 month after surgery (*P* > 0.05).

**Conclusion:**

Stereotactic minimally invasive puncture combined with urokinase for the treatment of small- and medium-volume hemorrhage in the basal ganglia, including catheterization through the long axis of the hematoma, led to significantly better drainage effects and fewer complications. However, there was no significant difference in short-term NIHSS scores between the two types of catheterization.

## 1. Introduction

Stroke is currently regarded as the second largest contributor to global disability-adjusted life years (DALYs) in developing countries (1). Although spontaneous intracerebral parenchymal hemorrhage (IPH) accounts for <20% of strokes, this condition is associated with high rates of morbidity and mortality. The bleeding site is usually located in the deep gray matter, including the basal ganglia and the thalamus (2). The recent results arising from the minimally invasive surgery plus alteplase for cerebral hemorrhage (MISTIE) III trial showed that minimally invasive surgery is safer than drug therapy and that thrombolysis after minimally invasive catheter evacuation reduces the size of hematomas by up to 15 ml, thus reducing mortality and improving prognosis (3). The MISTIE II trial demonstrated that the effects of surgery were directly associated with the position of the catheter. In this trial, the catheter was positioned along the entire longitudinal axis of the hematoma (defined as at least two-thirds of the longitudinal length). Stereotactic techniques for the placement of the drainage tube are known to be more accurate and controlled in the treatment of small-volume hemorrhage in the basal ganglia and have achieved effective outcomes (4, 5). Therefore, the purpose of this manuscript is to explore the effect of different catheter placement positions on the treatment of moderate to small amounts of hypertensive intracerebral hemorrhage.

## 2. Objects and methods

### 2.1. Research objects

In this trial, we recruited 95 neurosurgery patients from Yichang Three Gorges Central People's Hospital between June 2019 and March 2022 to receive stereotactic minimally invasive puncture and catheter placement combined with urokinase for the treatment of medium-to-small hemorrhages in the basal ganglia. Of these, 12 patients were surgical patients in the early stage of hemorrhages, and 83 patients were in the later stage of hemorrhages. The patients were divided into two groups by using a randomized number table method. According to the location of the catheter, the patients were divided into two groups as follows: those who were catheterized through the long axis of the hematoma (42 cases through the frontal approach) and those who received catheterization through the center of the hematoma (41 cases through the frontal–parietal approach). This trial complied with the relevant ethical standards and was approved by our hospital's ethics committee (KY-2022-0040). Signed and informed consent was obtained from all the patients or their legal representatives. The inclusion criteria were as follows: (1) all patients who were diagnosed with basal ganglia hemorrhage by head Computed Tomography (CT) and Computed Tomography Artery (CTA) examination on admission, according to the Guidelines for the Diagnosis and Treatment of Cerebral Hemorrhage in China (2019); (2) patients with a history of hypertension; (3) patients affected by basal ganglia hemorrhage for the first time and having no previous neurological dysfunction; and (4) patients with a bleeding volume in the basal ganglia of 20–40 ml. The exclusion criteria were as follows: (1) patients with severe coagulation dysfunction or severe basic diseases; (2) patients with surgical contraindications; (3) patients whose hemorrhage had broken into ventricles; and (4) patients whose hemorrhage was caused by brain tumors, cerebral aneurysms, cerebral vascular malformations, and other reasons (6).

### 2.2. Methods

We collated a range of general clinical data for each patient (Table 1), including gender, age, hematoma volume at admission, time from onset to visit, blood pressure at admission, Glasgow Coma Scale (GCS) at admission, smoking history, compliance with hyperlipidemia drugs, antiplatelet therapy, diabetes, hypertension, random blood pressure, other cardiovascular diseases, NIHSS score, and the time from stroke to first CT at admission.

**Table 1:** Clinical data of 83 patients with a medium-to-small basal ganglia hemorrhage.

**Items**	**Penetrating hematoma long-axis group (*n =* 42)**	**Hematoma center group (*n* = 41)**	***P*** **(group penetrating the long axis of hematoma vs. group at the center of hematoma)**
Gender (Men, %)	57.14%	48.78%	0.445^a^
Age (x ± s, age)	61.50 ± 8.18	64.04 ± 6.62	0.123^b^
Smoking (case, %)	26.19%	17.07%	0.314^a^
Diabetes (case, %)	9.52%	12.12%	0.696^a^
Hyperlipidemia (case, %)	66.67%	53.66%	0.226^a^
Use of Bayaspirin (case, %)	7.14%	9.76%	0.668^a^
History of cardiovascular disease (case, %)	11.90%	12.20%	0.968^a^
NIHSS score on admission (x ± s, score)	12.69 ± 3.69	12.14 ± 2.82	0.754^b^
GCS score on admission (x ± s, score)	10.98 ± 1.55	11.41 ± 1.96	0.262^b^
3-8 score (case)	4	2	
9-12 score (case)	26	27	
13-15 score (case)	12	12	
First CT bleeding volume (x ± s, ml)	26.40 ± 3.34	25.17 ± 3.68	0.114^b^
Preoperative CT bleeding volume (x ± s, ml)	26.64 ± 3.48	25.41 ± 3.78	0.128^b^
Time of admission after stroke (x ± s, hour)	2.91 ± 1.98	2.64 ± 1.79	0.520^b^
Time from stroke to operation (x ± s, hour)	21.98 ± 4.80	20.37 ± 4.71	0.127^b^
Systolic blood pressure at visit (x ± s, mmHg)	182.64 ± 8.61	181.12 ± 9.34	0.772^b^
Diastolic blood pressure at visit (x ± s, mmHg)	102.19 ± 4.67	101.31 ± 4.83	0.838^b^
Operative duration (x ± s, minute)	36.26 ± 5.21	35.82 ± 5.21	0.706^b^
Catheter accuracy (%)	97.62%	97.56%	0.986^a^

a chi-squared value,

b t value.

#### 2.2.1. Calculation of cerebral hemorrhage

According to the maximum level of hematoma selected by the CT cross-section, the longest diameter of the hematoma (cm) and the widest diameter of the hematoma (cm) were measured on the imaging system. The layer thickness was determined from the CT film, and the number of layers was defined as the total number of layers showing all hematomas. The volume of cerebral hematoma was calculated by the Toda formula (7), where hematoma volume (ml) = 1/2 × the longest diameter of the hematoma (cm) × the widest diameter of the hematoma (cm) × slice thickness (cm) × layers.

#### 2.2.2. The stereotactic minimally invasive catheter drainage surgery method

**Figure 1:**
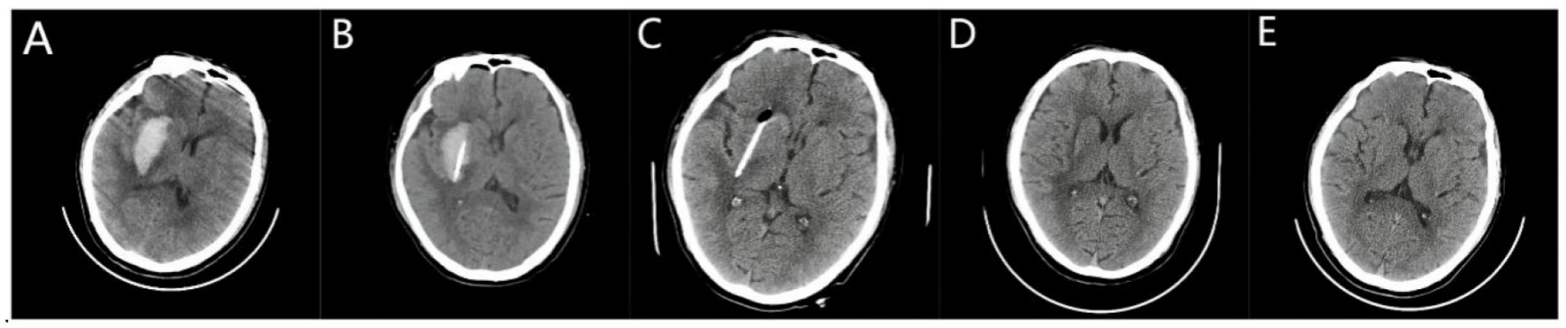
Stereotactic catheterization through the long axis of hematoma for basal ganglia hemorrhage. **(A)** A preoperative head Computed Tomography (CT) showed that the bleeding volume was ~29 ml. **(B)** A three-dimensional CT performed 6 h after surgery confirmed the position of the drainage tube along the long axis of the hematoma, with a hematoma volume of ~20 ml. **(C)** Following the injection of urokinase, the hematoma was completely drained by the third day after surgery. The high-density area indicates the drainage tube with a hematoma volume of 0 ml. **(D)** The bleeding site 7 days after surgery was slightly softer. **(E)** On the 20th day post-surgery, the softening lesion at the bleeding site was reduced.

**Figure 2:**
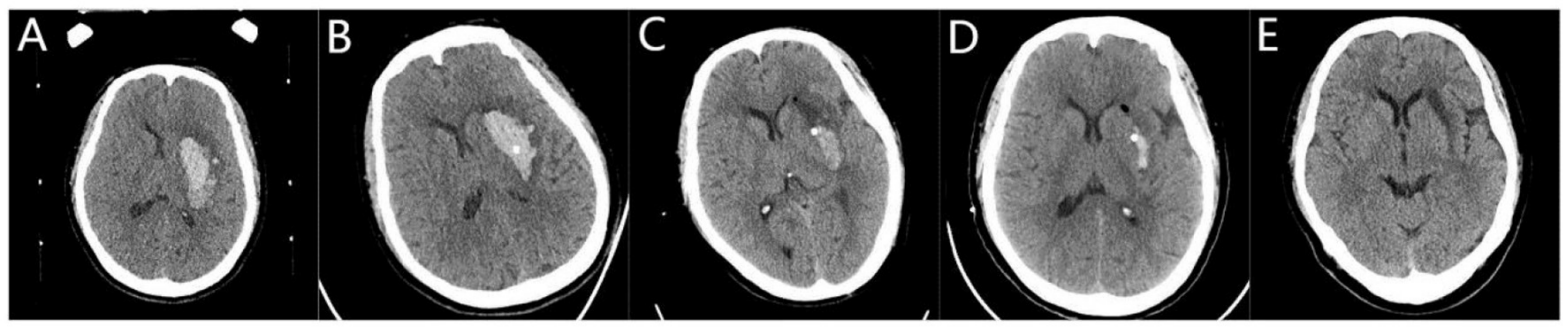
Stereotactic central catheterization for basal ganglia hemorrhage. **(A)** A preoperative head Computed Tomography (CT) showed that the volume of bleeding was ~26 ml. **(B)** A three-dimensional CT performed 6 h after surgery showed the drainage tube located in the center of the hematoma with a hematoma volume of ~17 ml. **(C)** Following the injection of urokinase, there was a small amount of residual hematoma on the third day after the operation, with a hematoma volume of 5 ml. **(D)** On the 7th day after surgery, the hematoma volume was 1.2 ml. **(E)** On the 20th day after surgery, the bleeding site softened.

Surgery can be completed using a frameless navigation system or a frame-based stereotactic system; in this trial, we used the Leskell G-frame system (Elekta Instrument AB, Box 7593,Kungstensgatan 18, SE-103 93 Stockholm, Sweden). Patients underwent surgery if CT reexamination indicated that the hematoma was stable after 6 h or more. All patients received stereotactic minimally invasive catheter drainage surgery. For patients in the long-axis group, the incision was 2–3 cm above the eyebrow arch on the same side of the hematoma and 2–2.5 cm beside the midline. The planned puncture approach and target point before surgery were through the hematoma (Figures 1A–E). The incision was made 9–11 cm above the eyebrow arch on the same side of the hematoma and 3–3.5 cm beside the midline for patients in the central group. The planned puncture approach and target were in the center of the hematoma before surgery (Figures 2A–E). The first step was to install a square Leksell headrest on the patient's head under local anesthesia. The second step was to perform 64 rows of Siemens head 3D CT scanning. Third, according to the preoperative surgical plan, we needed to directly measure and calculate the target coordinates (X, Y, and Z space coordinates) using a CT three-dimensional film reading system. Fourth, for patients in the long-axis group, we removed a fixed strut at the surgical side of the forehead; this action was not required for patients in the center group. The Leskell headstand was connected and fixed to a special neurosurgery operating table by an adapter. Next, surgery was performed under local anesthesia and ECG monitoring. The size of the incision was generally 2 cm. After drilling the skull, a 5-mm incision was made in the dura mater to avoid the excessive outflow of cerebrospinal fluid. The Leskell guide arc was then installed, and the drainage tube was inserted under the guidance of the stereotactic instrument to reach the target point. During surgery, 1 ml of negative pressure was used to draw ~5 ml of blood; the same volume of normal saline was injected for replacement. When the drainage was smooth, the surgical procedure was completed, and the drainage tube was led out through another subcutaneous tunnel.

#### 2.2.3. The urokinase injection method and extubation index

We performed another three-dimensional CT scan of the head 6 h after surgery to determine the position of the drainage tube, the size of the hematoma, and the extent of bleeding in the puncture tunnel. If the drainage tube position was satisfactory, we fully dissolved 50,000 units of urokinase in 5 ml of normal saline. After surgery, the dissolved urokinase was injected into the drainage tube 1–2 times a day, and the drainage tube was closed for 3 h before natural drainage. According to the residual situation of the hematoma, urokinase was injected multiple times into the hematoma cavity, as required. The CT reexamination showed that the drainage tube cannot effectively drain the hematoma, which was the extubation index. The catheterization time did not exceed 7 days.

#### 2.2.4. Observation index

We compared the general clinical data from the two groups of patients. A range of statistical data was collated, including the time of catheter insertion, the total dose of urokinase, the clearance rate of hematoma before extubation, the amount of residual hematoma, and the NIHSS score 1 month after surgery. We also recorded complications experienced by the two groups of patients during treatment, including intracranial infection, pulmonary infection, urinary system infection, secondary intracranial hemorrhage, lower limb vein thrombosis, stress ulcer, anemia, hypoproteinemia, and mortality within 1 month.

#### 2.2.5. Statistical analysis

The SPSS version 25.0 statistical software (IBM, International Business Machines Corporation, New York, USA) was used for data analysis. Measurement data were expressed as mean ± standard deviation (x ± s). Comparisons between groups of normally distributed data were performed using the independent sample *t*-test and analysis of variance (ANOVA). The Mann–Whitney U-rank sum test was used for the data that were not normally distributed. Numerical data were expressed as the number of cases and as percentages (%), and the data were compared using the chi-square test. A *P*-value of < 0.05 (bilateral) was considered to be statistically significant.

## 3. Results

### 3.1. Clinical materials

**Figure 3:**
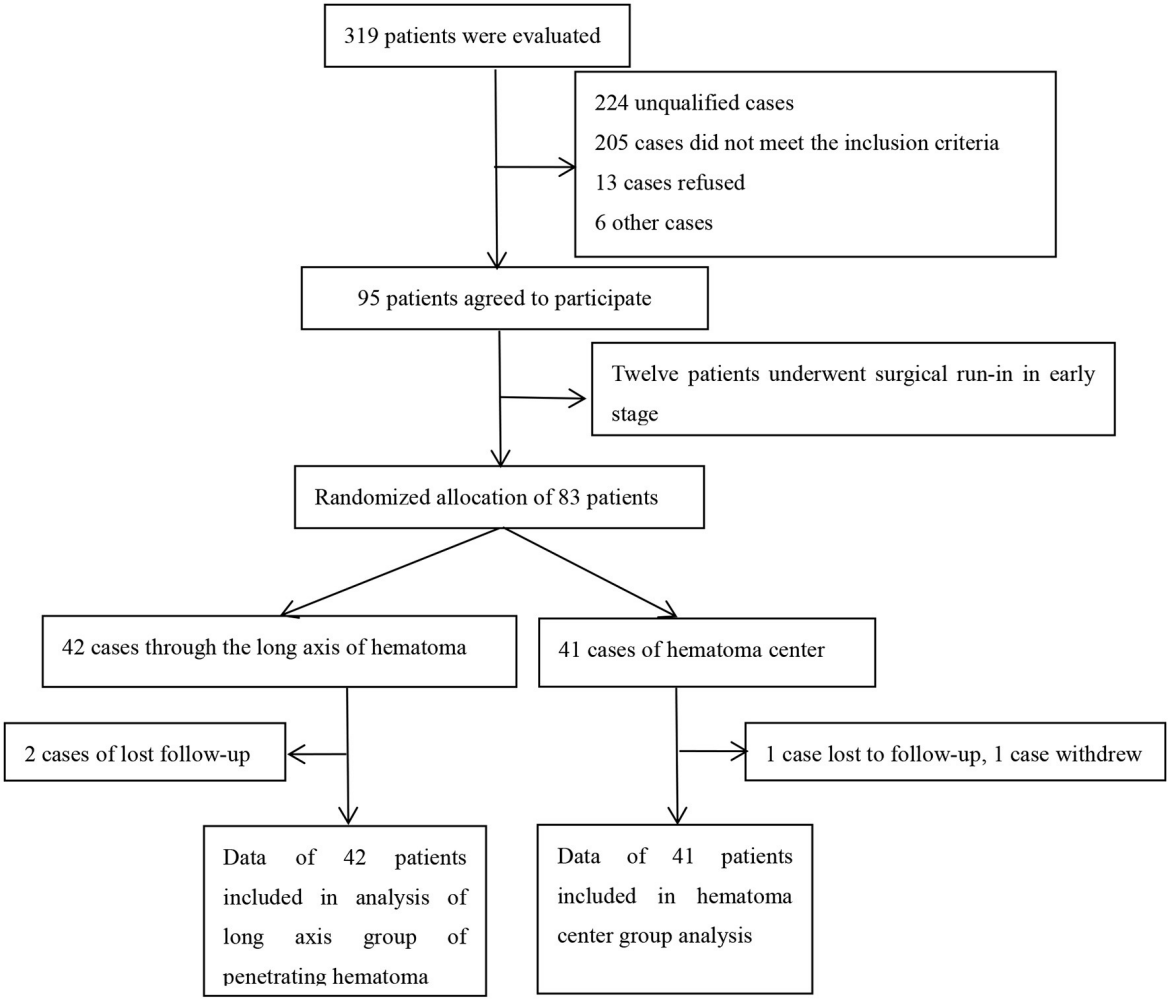
Overview of patient recruitment.

From June 2019 to March 2022, we recruited 95 patients, including 12 who underwent surgery in the early stage. In this trial, 83 patients with medium-to-small volumes of hemorrhage in the basal ganglia were included for final analysis (Figure 3). Of these patients, 42 of them were catheterized through the long axis of the hematoma, and 41 were catheterized through the center of the hematoma. Both patient groups were injected with urokinase through the drainage tube after surgery, as shown in Table 1.

### 3.2. A comparison of the hematoma drainage effect between the two groups

Compared with the central group, patients in the long-axis group had a significantly shorter catheterization time, a lower dose of urokinase, less residual hematoma, and a higher hematoma clearance rate (*P* < 0.05). There was no significant difference between the two groups in terms of NIHSS scores 1 month after surgery (*P* > 0.05) as shown in Table 2.

**Table 2:** Comparison of the hematoma drainage effect between the two groups (x̄ ± s).

	**Penetrating hematoma long-axis group**	**Hematoma center group**	***T*** **-value**	***p***
Tube setting time (d)	4.29 ± 0.60	4.65 ± 0.53	−3.009	0.003
Total urokinase (ten thousand U)	22.97 ± 4.82	27.92 ± 5.12	−4.536	0.000
Residual amount of extubation hematoma (ml)	1.31 ± 1.22	2.34 ± 1.49	−3.452	0.001
NIHSS score a month after operation	7.81 ± 2.21	8.66 ± 2.02	−1.825	0.072
Hematoma clearance rate (%)	93.71 ± 4.33	90.83 ± 5.80	2.564	0.012

### 3.3. A comparison of complications between the two groups during treatment

There was no significant difference between the two groups in terms of pulmonary infection, intracranial hemorrhage, urinary system infection, stress ulcer, anemia, and mortality within 1 month of surgery (*P* > 0.05). Compared with the central hematoma group, the incidence of intracranial infection, deep vein thrombosis, and hypoproteinemia in the long-axis group was significantly lower (*P* < 0.05), as shown in Table 3.

**Table 3:** Comparison of complications between the two groups during treatment (%).

	**Penetrating hematoma long-axis group *n* = 42**	**Hematoma center group *n* = 41**	***x*** **^2^ value**	***p***
Intracranial infection (case, %)	0%	9.76%	4.305	0.038
Pulmonary infection (case, %)	19.05%	17.07%	0.055	0.815
Secondary intracranial hemorrhage (case, %)	4.76%	2.44%	0.321	0.571
Urinary system infection (case, %)	11.90%	21.95%	1.493	0.222
Stress ulcer (case, %)	7.14%	12.20%	0.608	0.436
Deep vein thrombosis (case, %)	9.52%	26.83%	4.196	0.041
Anemia (case, %)	19.05%	29.27%	1.185	0.276
Hypoalbuminemia (case, %)	14.29%	34.15%	4.474	0.034
Mortality rate within a month (case, %)	0%	2.44%	1.037	0.309

## 4. Discussion

Hypertensive intracerebral hemorrhage (HICH) accounts for 21–48% of all patients with stroke. The most common type of HICH is basal ganglia hemorrhage (8), which accounts for 50% of patients with cerebral hemorrhage. In addition, patients often experience symptoms such as hemianopsia, hemiplegia, and hemiparesia due to damage to the cystic conduction tract in the basal ganglia region. It is very important to evaluate the neurological function of patients with cerebral hemorrhage in a timely fashion and determine whether surgical treatment is needed. There is no definite conclusion on the effects of surgical treatment for supratentorial intracerebral hemorrhage, although surgery is an option for patients with a large hematoma, with severe neurological dysfunction, or in coma. The surgical methods involve craniotomy and minimally invasive surgery. The mortality rate associated with craniotomy is very high. The surgical trials in intracerebral hemorrhage (STICH) and STICH II trials showed that patients with spontaneous supratentorial intracerebral hemorrhage did not have any overall benefit from early surgery when compared with conservative treatment (9, 10). However, 98% of patients in the STICH study underwent craniotomy; the researchers thus proposed that minimally invasive technology is more conducive for deep brain hematoma. Studies have reported that minimally invasive surgery is better than craniotomy (11–13). Minimally invasive surgical methods involve keyhole craniotomy, neuroendoscopy hematoma removal, and minimally invasive catheter drainage. In the MISTIE II trial, a stereotactic catheter was used for catheter placement and continuous thrombolysis; this method achieved accurate catheter placement. This procedure is highly reproducible and can safely and accurately drain a cerebral hemorrhage; the effects of hematoma drainage are closely associated with the accuracy of catheter placement. Currently, most minimally invasive catheters for intracerebral hemorrhage are placed at the midpoint of the largest hematoma layer in the brain as the puncture sites (14–16). However, few studies have investigated the effects of different catheter positions on hematoma drainage. In this study, we describe the results of our recent trial that examined the effects of different catheter positions for intracerebral hemorrhage.

### 4.1. The application of stereotactic minimally invasive catheterization technology for medium- and small-volume cerebral hemorrhage

The target error for stereotactic technology was previously determined to be 2.02 mm (17). This type of technology is widely adopted in minimally invasive neurosurgery, such as deep brain electrical stimulation in Parkinson's disease, epileptic electrode implantation, brain tumor biopsy, stereotactic puncture, and the aspiration of cerebral hemorrhage (18, 19). Compared with conservative treatment, the rebleeding rate of minimally invasive stereotactic catheterization in 20–40 ml HICH was significantly lower than that for conservative medical treatment. Furthermore, a hematoma can be cleared quickly, thus reducing compression caused by the hematoma on the brain tissue and the risk of secondary damage; this is conducive to the recovery of a patient's neural function, thus reducing hospital stay (20). In previous studies, when the amount of cerebral hemorrhage was >30 ml, craniotomy or endoscopic surgery was mostly used. Compared with craniotomy, stereotactic catheterization for an intracerebral hemorrhage of more than 30 ml is safe and effective and has fewer complications (21). The clinical effect of this treatment is better than that of traditional craniotomy and can thus avoid secondary cranioplasty. In our research, we found that the hematoma volume was more than 30 ml in some patients and 20–30 ml in others. The preoperative hematoma volume was 26.64 ± 3.48 ml in the long-axis group and 25.41 ± 3.78 ml in the central group. We found a relationship between hematoma volume and the enrollment data. Currently, minimally invasive surgery for intracerebral hemorrhage is regarded as a trend, but there is no specific standard for endoscopic surgery and stereotactic minimally invasive puncture (19). For 40–60 ml of bleeding, we prioritize craniotomy or endoscopic hematoma evacuation. Endoscopic intracerebral hemorrhage clearance has also advanced significantly. Endoscopic surgery can enhance the neurological function of patients with more than 40 ml of intracerebral hemorrhage at 6 months after surgery and can reduce the mortality of patients with a GCS score of 3–8 (22). Endoscopic surgery can quickly remove a hematoma, although the trauma incurred is relatively significant, thus requiring high levels of surgical skill. Moreover, the operation takes a long period and requires general anesthesia. Stereotactic minimally invasive catheterization is minimally invasive and can be performed under local anesthesia. Furthermore, the surgical time is reduced, and the operation is reproducible; however, the disadvantage of this technique is that a hematoma cannot be quickly removed, and the rate of rebleeding can be high (23). Stereotactic guidance can clear a hematoma along the long axis of the hematoma, combined with endoscopic removal of the intracerebral hemorrhage. Most blood clots can be cleared only once through the endoscopic sheath; this can minimize damage to normal brain tissue (8). Some studies have shown that the disability rate associated with stereotactic catheterization for 20–50 ml superficial and deep hemorrhage is lower than that of small bone window craniotomy and endoscopic surgery (19). Some elderly patients have a strong tolerance to increased intracranial pressure due to the presence of brain atrophy. We have attempted to use stereotactic minimally invasive catheterization in elderly patients with cerebral hemorrhage volumes exceeding 40 ml. Currently, only 10 cases of surgery have been attempted, although the final treatment effect is very good.

### 4.2. Selection of the best puncture path and target for stereotactic minimally invasive catheterization

In the MISTIE III trial, three methods of catheterization were adopted. The first method was to cut the forehead and insert a catheter along the longitudinal axis of the hematoma. The second method was to cut the parietal–occipital part and place a tube along the longitudinal axis of the hematoma. The third method was to cut the temporal part and place a tube along the transverse axis of the hematoma (3). The highest proportion of patients with hematomas smaller than 15 ml was the highest (79.3%) at the end of tube insertion when using the first method. When the drainage tube is located in the center of the hematoma, theoretically, it can make urokinase contact the hematoma more evenly, thus improving the dissolution effect of the hematoma; this can improve the hematoma clearance rate when the tube is withdrawn (24). In fact, in most cases, we found that when the catheter was placed in the center of the hematoma, although the effect of early hematoma dissolution was very good, there was a residual hematoma at the back when the catheter was removed. In our 41 patients, we found that the hematoma clearance rate was 90.83 ± 5.80%. We analyzed the causes of residual hematoma in the posterior regions. Most patients with intracerebral hemorrhage had their heads raised by 30° during surgery. Due to positional factors and the gravity of the brain tissue itself, the hematoma around and in front of the drainage tube hole can often provide easy contact with urokinase. Most of the hematomas were successfully drained, thus resulting in the drainage tube hole being located at the edge of the posterior residual hematoma. Without further surgery, the drainage tube hole cannot be adjusted to contact the hematoma again; therefore, the urokinase injected through the catheter cannot fully contact the posterior hematoma because of the residue. When inserting the tube through the long axis of the hematoma, the catheter hole is located at the rear of the hematoma, and the urokinase can dissolve the hematoma around the drainage tube hole (25). The direction of hematoma dissolution occurs from back to front. When the current hematoma dissolution and drainage were poor, we rechecked the CT to investigate the relationship between the drainage tube and the hematoma. Because the tube is placed through the long axis of the hematoma, this was performed by withdrawing part of the drainage tube to make the tube hole make full contact with the hematoma again, thus reducing residuals. The clearance rate of hematoma for the 42 patients was 93.71 ± 4.33%. In four cases, the drainage tube was partially withdrawn to achieve a satisfactory drainage effect. According to the MISTIE III trial and other research results, we recommend transfrontal long-axis catheterization for hematomas. This method has a good drainage effect, can reduce the number of urokinase injections, and can reduce the risk of intracranial infection.

### 4.3. Analysis of residual hematoma volume, patient prognosis, and complications

The survival rate of patients with intracerebral hemorrhage and the recovery of neurological function are related to the location of the hematoma, space-occupying effects, and intracranial pressure; these factors are also related to the long-term neurological dysfunction caused by neurotoxicity or inflammatory brain edema around the hematoma (26, 27). Numerous preclinical and clinical studies have shown that perihematoma edema (PHE) is a quantifiable marker for secondary brain injury after intracerebral hemorrhage (ICH) and is associated with poor prognosis (28). In ~30% of patients, the volume of PHE 2–3 weeks after ICH was 3 ml larger than that 1 week after ICH; this increase in volume was reported to represent an independent risk factor for poor prognosis (29). Minimally invasive surgery may change the course of PHE. The extent of edema in PHE was associated with hematoma clearance, and a higher percentage of hematoma clearance has been shown to result in a slower increase in PHE (30). In a previous study, 28% of patients with a residual hematoma volume of >15 ml had an Modified Rankin Scale (mRS) of ≤ 3 at 180 days, and 49% of patients with a residual hematoma volume of ≤ 15 ml had an mRS of ≤ 3 at 180 days (31). The increase in PHE at 72 h may have had adverse effects on the neurological recovery of ICH, especially in patients with small-to-medium volume hematomas (32). Increasing the clearance rate of residual hematomas after surgery is conducive to reducing brain injury after ICH (33). Our study showed that there was no significant difference in short-term neurological recovery between the two groups; this may be related to the reduction in a residual hematoma or the short follow-up time of the two groups of patients during extubation. There was a certain difference in the residual amount of hematoma between the two different methods of catheter insertion, although the hematoma was < 15 ml in volume when the catheter was removed. Common medical complications after intracerebral hemorrhage include pneumonia (24%), acute renal injury (9%), ventricular inflammation (5%), asymptomatic rebleeding (4%), ischemic stroke (3%), surgical infection (1%), and others (31, 34). In the acute phase of intracerebral hemorrhage, the reduction in hematoma volume is conducive to reducing potential medical complications (35). Our study also confirmed that patients with a higher hematoma clearance rate had fewer complications in the short term.

### 4.4. Advancing the accuracy of stereotactic catheterization and reducing surgical-related complications

Because the stereotactic technique was not adopted in the MISTIE III trial, the rate of good catheter placement was 62%, although 6.8% of patients with poor catheter placement needed replacement of the drainage tube. We adopted the stereotactic technique to insert the catheter in 83 cases; proper placement of the drainage tube was achieved in 97.59% of cases. No cases required replacement of the drainage tube; this instance depends on the improvement of the method used to replace the catheter during surgery. To improve the accuracy of stereotactic catheterization, the following points should be considered: (1) avoiding the implantation of soft drainage tubes through the broken suction needle path in the hematoma; (2) selecting a drainage tube that matches the aperture of the guide sub; (3) directly using a soft drainage tube with a hard tube core for puncture; (4), when the drainage tube is located at the target position, the first suction of blood does not exceed 5 ml; and (5) adjusting the position of the Leksell head frame bone nail according to the hematoma location to avoid the influence of artifacts of skull bone nails during CT scanning. The following points should also be considered to reduce surgical-related complications: (1) reducing the number of urokinase injections can reduce the risk of infection; (2) as some patients may experience complete drainage of a hematoma on the second day, it is important to avoid removal of the drainage tube at this time. Removal of the drainage tube too early may increase the risk of bleeding; (3) the retention time of the drainage tube shall not exceed 7 days. Even if there is residual hematoma at this time, the drainage tube should be removed. If the drainage tube is left for too long, it will increase the risk of intracranial infection; and (4) if the costs are not considered, we would recommend the use of alteplase as this drug has a lower risk of bleeding. However, the clinical application of alteplase has proven that it is safe, effective, and controls the amount of urokinase used for each hematoma dissolution (36).

For the surgical treatment of small and medium volumes of ICH in the basal ganglia, patients can undergo stereotactic catheter drainage (37). We found that the placement of the catheter was accurate, and the residual amount of hematoma after drainage was < 15 ml. There was no significant difference in short-term neurological function scores between hematoma long-axis catheterization and hematoma central catheterization. However, we recommend inserting a tube through the long axis of the hematoma through the forehead approach, because the hematoma clearance rate is high and the dose of urokinase required is small, thus reducing medical complications. One limitation of this study is that it was a single-center study with a small number of cases and a short follow-up time. Currently, multicenter case data are being collected for further analysis.

## Data availability statement

The raw data supporting the conclusions of this article will be made available by the authors, without undue reservation.

## Ethics statement

The studies involving human participants were reviewed and approved by Human Ethics Committee of People's Hospital of China Three Gorges University (KY-2022-0040). The patients/participants provided their written informed consent to participate in this study. Written informed consent was obtained from the individual(s) for the publication of any potentially identifiable images or data included in this article.

## Author contributions

LJ and ZY prepared Tables 1, 2. All authors made substantial contributions to the conception and design, the acquisition of data, analysis and interpretation of data, drafting, critical revision, and approval of the final version of this manuscript.
